# Society for Endocrinology guidelines for the diagnosis and management of post-bariatric hypoglycaemia

**DOI:** 10.1530/EC-23-0285

**Published:** 2024-04-01

**Authors:** Jonathan Hazlehurst, Bernard Khoo, Carolina Brito Lobato, Ibiyemi Ilesanmi, Sally Abbott, Tin Chan, Sanesh Pillai, Kate Maslin, Sanjay Purkayastha, Barbara McGowan, Rob Andrews, Eveleigh Nicholson, Katherine McCullough, Lorraine Albon, Rachel Batterham, Georgios K Dimitriadis, Shareen Forbes, Gavin Bewick, Tricia M-M Tan

**Affiliations:** 1 Department of Diabetes and Endocrinology, University Hospitals Birmingham NHS Foundation Trust, Birmingham, UK; 2 Endocrinology, Division of Medicine, University College London, London, UK; 3 Department of Biomedical Sciences, Faculty of Health and Medical Sciences, University of Copenhagen, Copenhagen, Denmark; 4 Department of Medicine, Copenhagen University Hospital – Amager and Hvidovre, Hvidovre, Denmark; 5 Section of Endocrinology and Investigative Medicine, Department of Metabolism, Digestion and Reproduction, Faculty of Medicine, Imperial College London, London, UK; 6 Department of Dietetics, University Hospitals Coventry and Warwickshire NHS Trust, Coventry, UK; 7 Faculty of Medicine, Chinese University of Hong Kong, Hong Kong; 8 Centre for Endocrinology, Diabetes and Metabolism, Birmingham Health Partners, Birmingham, UK; 9 School of Nursing and Midwifery, University of Plymouth, Plymouth, UK; 10 Brunel University, London, UK; 11 Imperial College Healthcare NHS Trust, St Mary’s Hospital, London, UK; 12 Endocrinology, Guys’ and St Thomas’s NHS Foundation Trust, London, UK; 13 University of Exeter Medical School, Exeter, UK; 14 Portsmouth Hospitals University NHS Trust, Portsmouth, UK; 15 Royal Surrey County Hospital, Guildford, UK; 16 University Hospitals Sussex NHS Foundation Trust, Worthing, UK; 17 King's College Hospital NHS Foundation Trust, London, UK; 18 BHF Centre for Cardiovascular Science, Queen’s Medical Research Institute, University of Edinburgh, Edinburgh, UK; 19 School of Life Course Sciences, Faculty of Life Sciences and Medicine, King's College London, London, UK

**Keywords:** obesity, bariatric surgery, post-bariatric hypoglycaemia, late dumping

## Abstract

Post-bariatric hypoglycaemia (PBH) is typically a post-prandial hypoglycaemia occurring about 2–4 h after eating in individuals who have undergone bariatric surgery. PBH develops relatively late after surgery and often after discharge from post-surgical follow-up by bariatric teams, leading to variability in diagnosis and management in non-specialist centres.

**Aim:**

The overall aim was to improve and standardise clinical practice in the diagnosis and management of PBH. The objectives were: (1) to undertake an up-to-date review of the current literature; (2) to formulate practical and evidence-based guidance regarding the diagnosis and treatment of PBH; (3) to recommend future avenues for research in this condition.

**Method:**

A scoping review was undertaken after an extensive literature search. A consensus on the guidance and confidence in the recommendations was reached by the steering group authors prior to review by key stakeholders.

**Outcome:**

We make pragmatic recommendations for the practical diagnosis and management of PBH, including criteria for diagnosis and recognition, as well as recommendations for research areas that should be explored.

**Plain English summary:**

Post-bariatric hypoglycaemia (PBH) is a condition that commonly affects people who have undergone weight loss surgery. In this condition, people develop low blood sugar occurring about 2–4 h after meals, leading to debilitating symptoms such as hunger, sweating, anxiety, palpitations and even blackouts and fainting. PBH is becoming more common as weight loss surgery is being taken up by more people to help with their weight and to help with diabetes. The condition often develops after the patient has been discharged from follow-up after their surgery, which can lead to inconsistent diagnosis and treatment in non-specialist healthcare centres. The lack of clear information and evidence in the existing scientific literature further contributes to the variation in care. To address this problem, the Society for Endocrinology has created new guidelines to help healthcare professionals accurately diagnose and manage this condition. The guidelines were developed with input from dietitians, surgeons and doctors specialising in weight loss, and hormone specialists.

## Introduction

Bariatric surgery, such as Roux-en-Y gastric bypass, gastric banding, or sleeve gastrectomy, carries significant metabolic benefits, including weight loss, diabetes remission, and reduction of macrovascular and microvascular events. As a result of this clinical evidence, bariatric surgery is more often considered as an option for the treatment of obesity and diabetes, as well as for other metabolic complications such as non-alcoholic fatty liver disease (1). Given the increasing prevalence of bariatric surgery, clinicians are increasingly encountering complications of surgery (2). One such complication is post-bariatric hypoglycaemia (PBH) (3). PBH is usually defined as post-prandial hyperinsulinaemic hypoglycaemia, occurring typically 2–4 h after ingestion of food, and presenting with documented low glucose values and hypoglycaemic symptomatology, which subsides once euglycaemia is restored – i.e. Whipple’s triad.

PBH is associated with a lower quality of life, which improves after treatment (4). Hypoglycaemia leads to psychological consequences, prolonged disruption of work and leisure activities, the possibility of road traffic accidents and limitations to the types of work that can be taken up (5). Moreover, recurrent hypoglycaemia can lead to impaired awareness of hypoglycaemia, impaired cognitive function, cardiac arrhythmia and mortality (6). As a result, it is important to identify, diagnose, and adequately treat PBH in people who have undergone bariatric surgery.

## Aims and objectives

The aim of this guideline is to improve and standardise clinical practice in the diagnosis and management of PBH. To this end, our objectives were to: (1) undertake an up-to-date review of the current literature; (2) formulate practical and evidence-based guidance regarding the diagnosis and treatment of PBH and (3) recommend future avenues for research in this condition.

## Materials and methods

The Society for Endocrinology’s Clinical Committee commissioned this guideline and appointed TT and JH as leads of the guideline committee. TT and JH nominated an initial working group (JH, TT, II, SP, TC and BK). Meetings were held virtually to assign specific areas of the guideline group to produce reports based on their own narrative reviews and a synthesis of the wider literature that JH and TC were tasked to provide. The initial working group and subsequent wider expert panel group provided peer review of the reports and recommendations.

JH and TC undertook a detailed search to synthesise the relevant clinical literature as well as identify prior guidance and recommendations for the diagnosis and management of PBH. This took the form of a broad scoping exercise to identify the relevant literature. The initial working group contributed to search terms with the search run through the Ovid platform for MEDLINE and EMBASE as well as a separate search in the Cochrane Database. The scoping exercise was pre-registered on the OSF open science platform (https://osf.io/) and discussed at the National Obesity Update meeting as well as the Metabolic Endocrinology Network within the Society for Endocrinology. Examples of the search terms are included (Appendix 1, see section on supplementary materials given at the end of this article). The search was initially run on 17 November 2020 and updated on 31 January 2022. Abstracts were further reviewed to include relevant clinical human literature with the exclusion of single case reports. Conference abstracts were included to broaden the identification of relevant literature. The synthesised literature was then checked and assembled by the core writing team of BK, JH and TT. The co-chairs and the wider group were able to reach a consensus that underpins the guideline. The confidence in each recommendation was assessed and graded using GRADE criteria (7). An advanced draft of the guideline was reviewed by the Society for Endocrinology Clinical Committee before submission for publication. Further information and clarification were added with the assistance of BK, TT and CBL after peer review.

## Pathophysiology of PBH

The exact pathophysiology of PBH is unknown, but the dynamic interaction of food absorption, incretin, and insulin secretion is thought to play a role. Following most types of bariatric surgery, with the exception of gastric banding, there is a rapid transit of nutrients to the jejunum and the ileum with absorption and a rapid peak of glucose, which provokes insulin secretion. The exposure of L cells in the distal jejunum and proximal ileum to these nutrients also provokes the secretion of GLP-1 and other incretins, which amplify the hyperglycaemia-dependent secretion of insulin and the insulin-dependent disposal of glucose (8). A leading theory is that some people suffer from an exaggerated version of this incretin and insulinotropic response, leading to an ‘overswing’ hypoglycaemia, i.e. PBH (9). In support of this theory, a GLP-1 receptor antagonist (exendin 9–39) abolishes hypoglycaemia in people with PBH (also discussed below). Other possible mechanisms include impaired suppression of basal insulin secretion in response to hypoglycaemia and excessive meal-stimulated insulin secretion, defective alpha-cell secretion of glucagon (10, 11, 12, 13), alterations in bile acid kinetics (14) which may in turn trigger excess FGF-19 secretion (15), and inflammatory cytokines such as IL-1beta (16).

PBH is also known as ‘late dumping syndrome’. This phenomenon was first described in people after upper gastrointestinal surgery such as oesophagectomy, Nissen fundoplication (17), and gastrectomy for cancer (18). This contrasts with ‘early dumping syndrome’, which occurs within minutes of food ingestion. Early dumping is characterised by a combination of gastrointestinal symptoms (pain, bloating, nausea, diarrhoea and borborygmi) with vasomotor symptoms (fatigue, flushing, palpitations, sweating, tachycardia, hypotension and syncope) without hypoglycaemia. Early dumping syndrome is thought to be mediated by the arrival of a hyperosmolar meal in the small intestine, which in turn leads to a shift of fluid from the intravascular fluid compartment to the intestinal lumen, causing distension of the intestine, a reduction in intravascular fluid volume and stimulation of the autonomic and entero-neuro-endocrine systems (19). However, the distinction between ‘early dumping’ and ‘late dumping’/PBH may not be as clear as these definitions suggest. The two classes of dumping may co-exist and may be aetiologically interrelated.

## Prevalence of PBH

The true prevalence of PBH following bariatric surgery remains unknown and is confounded by differences in diagnostic methods/criteria (see below). It is estimated to be between 25% and 75% based on continuous glucose monitoring studies, 19–30% following provocative tests and using hypoglycaemia-related events. Registry-based studies report a far lower prevalence of 0.1–0.9% following bariatric surgery (20, 21, 22, 23).

## Risk factors for PBH

Currently, there are contrasting reports regarding the risk factors for PBH. Some observational studies have suggested that people who develop PBH are non-diabetic pre-operatively and have lower pre-operative HbA1c. This phenomenon is possibly related to higher pre-operative β-cell function, insulin secretion and sensitivity (24). Indeed, the presence of pre-operative hypoglycaemia during an oral glucose tolerance test (OGTT) is associated with PBH (25). Other possible risk factors are younger age, lower BMI, female gender, higher weight loss post-surgery (26, 27). However, other studies have found no association between hypoglycaemia and some of the abovementioned risk factors, such as magnitude of weight loss, age and gender, although the latter study relied on self-reporting of hypoglycaemia.

The type of bariatric surgery has been suggested to be a risk factor. People who have undergone laparoscopic adjustable gastric banding (LAGB) are at a low risk of PBH; however, this type of surgery is less performed at present due to possibly lower efficacy than RYGB and SG (28). The risk of PBH appears to be similar between RYGB and SG, although there is a higher rate of severe PBH with hospitalisation after RYGB (29). A study comparing the occurrence of hypoglycaemia after OGTT between age, sex and preoperative BMI-matched people who had undergone RYGB, SG, biliopancreatic diversion (BPD) and single anastomosis duodenal-ileal bypass with sleeve gastrectomy (SADI-S) showed lower rates of post-OGTT hypoglycaemia in the SADI-S and BPD groups (30). Roslin *et al.* reported that people after RYGB reported more hypoglycaemic symptoms than duodenal switch (DS, often combined with BPD) or SG (31). The prevalence of hypoglycaemia after single-anastomosis gastric bypass (SAGB, also known as one-anastomosis or ‘mini’ gastric bypass) has not been studied systematically with conflicting data. Two surgical series reported no hypoglycaemia, whereas another series reported that both RYGB and SAGB were equal with respect to PBH but did not quantify this statement (32). Another series of PBH people included those who had undergone SAGB, and one study showed an increase in time in hypoglycaemia on continuous glucose monitoring after both RYGB and SAGB (33). Therefore, it is likely that SAGB is also associated with PBH, but it is not presently clear whether this surgery carries any benefits for PBH relative to RYGB.

There is a suggestion from work done by one group that cholecystectomy may increase the risk of PBH, perhaps by altering the kinetics of bile acid absorption after eating, but this observation has yet to be confirmed by other groups.

## Diagnosis

**Figure 1:**
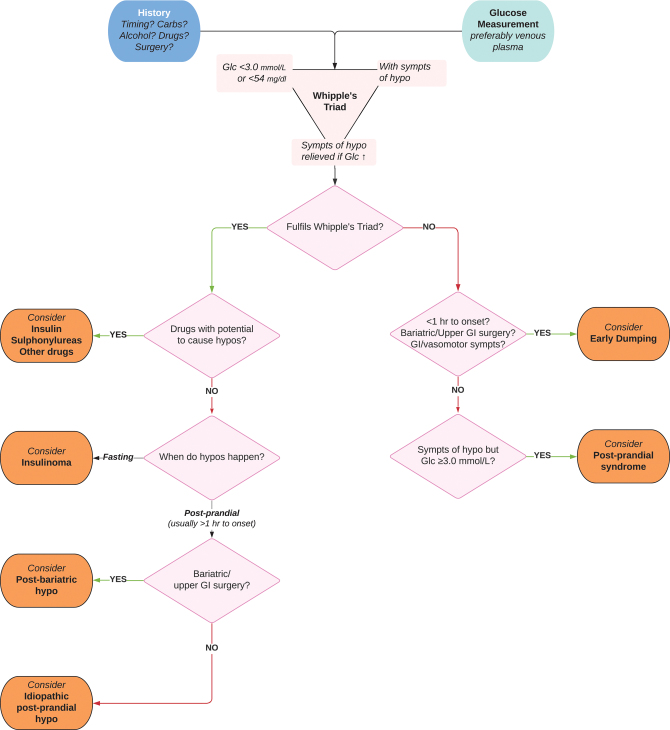
Framework for diagnosis of post-bariatric hypoglycaemia and allied conditions.

A thorough history, including onset of symptoms after surgery and timing of hypoglycaemia and food intake, should be taken (Fig. 1; Recommendation 1.1, Table 1). Investigations should proceed when the components of Whipple’s triad are present:

**Table 1:** Summary table of recommendations. Confidence in guidance recommendations classified using GRADE (7).

**1.**	**Diagnosis**	**GRADE certainty**
1.1	People potentially presenting with PBH should undergo a careful initial evaluation including consideration of alternative causes of hypoglycaemia.	N/A
1.2	PBH should be defined pragmatically in a patient after bariatric surgery as biochemically confirmed hypoglycaemia <3.0 mmol/L (54 mg/dL), with typical hypoglycaemic symptoms, fulfilling Whipple’s triad, after investigation and exclusion of alternative causes of hypoglycaemia.	Moderate
1.3	Hypoglycaemia should preferably be confirmed in a venous plasma sample utilizing an assay that is validated for low glucose concentrations.	High
1.4	Dynamic or provocational testing with OGTT or MMT is not recommended for the diagnosis of PBH.	Moderate
1.5	CGM is not presently recommended for the diagnosis of PBH.	Moderate
1.6	If a venous plasma glucose measurement is not available or practical, capillary blood glucose measurements can also be acceptable for diagnosis if they are taken using a testing system which conforms to ISO 15197 standards, there are multiple confirmatory readings and the history is strongly consistent with PBH.	Low
**2.**	**Behavioural, dietary and lifestyle management**	
2.1	People with PBH should undergo evaluation for dietary habits, dietary triggers and eating behaviours by a registered dietitian, preferably with a specialist interest in bariatric surgery, to enable individualised guidance on a healthy post-bariatric eating strategy.	Moderate
2.2	People with PBH should also be screened and treated for nutritional deficiencies according to accepted guidance.	High
2.3	Dietary advice for PBH should include eating controlled portions of carbohydrates, together with reinforcement of the post-bariatric eating strategy. Suggested strategies include: - Small and frequent meals (up to 6× per day). - Reduced carbohydrate content (starting at <30 g/meal). - Emphasis on choosing low GI over high GI carbohydrates. - Consideration may be given to lower carbohydrate targets (e.g. 25 g/meal, 20 g/meal and so on) under supervision if the initial 30 g/meal target is not effective.	Moderate
2.4	We do not recommend modifying the type of carbohydrates (e.g. fructose-containing foods), using cornstarch supplements, or using food additives to modify glycaemic index.	Low
2.5	CGM, where available or funded, may be considered as a tool for education, reinforcing and documenting the relationship between dietary changes to the amelioration of PBH, improving the adherence to dietary advice and for early recognition and treatment of hypos.	Low
2.6	People with PBH should be taught procedures to treat emergent hypoglycaemia. A suggested approach is as follows: - They should be advised to always carry fast acting carbohydrates to treat hypoglycaemia. - Upon receiving a warning or confirmation of hypoglycaemia, they should be advised to consume small amounts of fast acting carbohydrates (e.g. 10–15 g of dextrose). - They should be advised to test blood glucose 15 min after consumption to confirm resolution of the hypoglycaemia. If necessary, a further small dose of carbohydrate can be used if the blood glucose is still low. - A follow-on low GI carbohydrate-containing snack may be considered after initial resolution of the hypoglycaemia.	Low
2.7	Sucrose for treatment of hypoglycaemia should be avoided in acarbose-treated PBH.	High
2.8	People should be encouraged to exercise after bariatric surgery. A suggested approach to exercise in PBH is as follows: - They should be advised to take exercise when the risk of PBH is low (e.g. when fasting or at least 3 hr after meals). - They should be advised to avoid carbohydrate loading prior to exercise. - They should be advised to measure their blood glucose when feeling unwell. - They should be advised to carry some fast-acting carbohydrates that can be used to manage any hypoglycaemia. - A follow-on low GI carbohydrate-containing snack may be considered after exercise.	Low
2.9	People with PBH should be encouraged to reduce or cease alcohol intake.	Low
2.10	Modifications to dietary macronutrient content such as increased consumption of protein during meals should be encouraged. Individualised targets for protein consumption should be set. These are minimally 60 g/day but higher targets may be required.	Moderate
2.11	People with PBH should be encouraged to avoid fluids with each meal. Ideally, they should avoid drinking in the 30 min prior to and continuing for 30 min after each meal, and minimally they should avoid drinking with the meal and for at least 30 min after each meal.	Low
2.12	Gastrostomy feeding tube placement into the remnant stomach may be considered in severe PBH which does not respond to dietary nor pharmacological treatment.	Low
**3.**	**Pharmacological management**	
3.1	Acarbose should be considered as the first-line pharmacological treatment for PBH.	Moderate
3.2	Second-line options, if acarbose is not effective or not tolerated, include short-acting SC pasireotide or octreotide (given three times a day before meals) or long-acting IM pasireotide LAR.	Moderate
3.3	Diazoxide may be considered as a third-line option if somatostatin analogues are not tolerated.	Low
3.4	In people with frequent, severe PBH, consider prescribing glucagon injections (GlucaGen®, Ogluo®) as rescue treatment, with appropriate training for helpers.	Moderate
3.5	Avexitide (exendin 9–39) may be considered as part of investigational trials for PBH, where available.	Moderate
3.6	We do not currently recommend long-term treatment of PBH with other types of long-acting somatostatin analogues such as IM octreotide LAR or lanreotide, SGLT inhibitors, GLP-1 analogues, IL-1beta antagonists, DPP-IV inhibitors and calcium channel antagonists.	Low
**4.**	**Surgical or endoscopic management**	
4.1	Decisions regarding reversal surgery should be discussed in a multidisciplinary setting, preferably with bariatric surgeons experienced in these techniques, and with full disclosure as to the likelihood of post-operative complications and weight regain.	N/A
4.2	Partial pancreatectomy may be indicated in cases where there is evidence of a coincident insulinoma/pancreatic mass and evidence of localised excess insulin secretion (e.g. from intraarterial calcium stimulation and hepatic vein sampling for insulin). Cases should be referred to experienced hepato-pancreato-biliary multidisciplinary teams.	Low
**5.**	**Driving in people diagnosed with PBH in the UK**	
5.1	All people diagnosed with PBH should be advised to contact the DVLA for advice.	
5.2	They should be advised to stop driving and contact the DVLA if they experience episodes of severe hypoglycaemia (defined as requiring the assistance of another person), or if there is evidence of impaired awareness of hypoglycaemia.	
5.3	People with PBH driving Group 1 vehicles (cars, motorcycles) can utilise glucose monitoring sensor systems but must carry back-up capillary blood glucose systems in their vehicles.	
5.4	People with PBH driving Group 2 vehicles (trucks, buses) must use back-up capillary blood glucose systems.	
**6.**	**Research area recommendations**	
6.1	The diagnostic performance and cost-effectiveness of CGM and capillary blood glucose in the diagnosis of PBH.	
6.2	The relationship of low interstitial glucose readings from CGM to the symptoms of PBH.	
6.3	The clinical efficacy and cost-effectiveness of utilising CGM for the management of PBH.	
6.4	The optimal method for treatment of hypoglycaemia in people with PBH.	
6.5	Controlled trials of potential treatments against the current standard-of-care (e.g. best dietary practice).	
6.6	The efficacy and long-term effects of alternative approaches such as endoscopic revision of gastro-jejunal anastomoses (TORe).	

CGM, continuous glucose monitoring; GI, glycaemic index; MMT, mixed meal test; OGTT, oral glucose tolerance test; PBH, post-bariatric hypoglycaemia..

- Symptoms of hypoglycaemia, such as sympathetic nervous system activation leading to tremor, palpitations, sweating, anxiety, and neuroglycopenic symptoms such as mood swings, irrational behaviour, confusion, weakness, seizures, decreased conscious levels, and syncope.
- Biochemically confirmed low blood glucose <3.0 mmol/L (<54 mg/dL), preferably utilising a venous plasma sample and an assay validated for low glucose concentrations. This cut-off is recommended in line with ADA and EASD recommendations, as blood glucose below this threshold is associated with impaired hypoglycaemia awareness, impairment of cognitive function, cardiac arrhythmia, and mortality (6).
- Relief of symptoms when hypoglycaemia is corrected.

Characteristically, PBH appears between 1 and 3 years after surgery and 2–4 h after eating. Physical activity may trigger these hypoglycaemic symptoms.

Differential diagnoses for PBH include the use of diabetes drugs such as insulin and sulphonylureas, other drugs such as quinine and hydroxychloroquine, causes of endogenous hyperinsulinaemia such as insulin- or proinsulin-secreting tumours, anti-insulin antibody syndrome and non-hyperinsulinaemic causes of hypoglycaemia such as hypoadrenalism, malnutrition, liver and kidney disease and sepsis. The workup for these differential diagnoses is outside the scope of this guidance, but internationally accepted guidance for this is available (34). PBH should also be distinguished from idiopathic postprandial syndrome, which is the occurrence of the symptoms of hypoglycaemia without actual hypoglycaemia, and early dumping syndrome (see above); however, as noted above, early and late dumping syndromes can co-exist.

### Oral glucose tolerance test

This diagnostic test has been the most widely studied in PBH, though a number of uncertainties relating to cut-offs, duration of testing, symptomatology during testing, and clinical utility remain (Table 2). The OGTT relies on a pure carbohydrate load, in liquid form, ingested by people within 5–10 min. The amount of glucose given varies, with most studies administering 75 g. Glucose, and where available, insulin and C-peptide, are assessed at baseline and up to 4 h after administration of glucose. The length of the test is variable, lasting up to 4 h. The definition of hypoglycaemia varies between studies. There is a high prevalence of induced hypoglycaemia when asymptomatic post-surgical cohorts are screened, suggesting a likely high false positive rate if used injudiciously. There is also a limited correlation between reported symptoms of subjective hypoglycaemia and biochemically confirmed hypoglycaemia following OGTT.

**Table 2:** Summary table of studies of OGTT in PBH.

Study	Population	Type of OGTT when test was done	Definition of hypoglycaemia	Prevalence of hypoglycaemia
Roslin, 2011 (35)	RYGB, *n*=36 Symptoms not stated	75 g OGTT over 4 h At least 6 months post-op	≤60 mg/dL (3.3 mmol/L) or drop of ≥100 mg/dL (5.6 mmol/L) over 1 h	72% of RYGB
Chen, 2013 (36)	SG, *n*=33 RYGB, *n*=96 Pre-existing T2DM symptoms not stated	OGTT stimulus not stated, length not stated pre-op, 12 weeks and 12 months post-op	Not stated	36.4% of SG 12.5% of RYGB
Itariu, 2014 (37)	RYGB, *n*=30 SG, *n*=3 LAGB, *n*=3 Symptoms not stated	75 g OGTT over 2 h Pre-op and approximately 12 months post-op	<60 mg/dL (3.3 mmol/L)	50% overall, all in RYGB group 53% of RYGB None for SG and LAGB
Capristo, 2018 (29)	RYGB, *n*=59 (4 symptomatic) SG, *n*=60 (all asymptomatic)	75 g OGTT over 3 h At 12 months post-op	<55.8 mg/dL (3.1 mmol/L)	29% of RYGB 14% of SG
Pigeyre, 2015 (38)	RYGB, *n*=222, (including those symptomatic of dumping or hypoglycaemia) LAGB, *n*=129 (all asymptomatic)	75 g OGTT over 2 h At 12 months post-op	<50 mg/dL (2.8 mmol/L)	10% of RYGB None for LAGB
Brix, 2019 (39)	RYGB, *n*=175 SG, *n*=62 LAGB, *n*=44 All asymptomatic	75 g OGTT over 2 h At 24 months post-op	<2.8 mmol/L	32.6% after RYGB 22.6% after SG 2.3% after LAGB
Guarino, 2019 (40)	RYGB, *n*=35 Pre-existing T2DM (11 had mod-severe symptomatic hypoglycaemia)	75 g OGTT over 3 h Pre-op and at 24 months post-op	≤60 mg/dL (3.3 mmol/L)	31% of RYGB
Papamargaritis, 2019 (41)	SG, *n*=18 (symptoms not categorised though scoring included)	75 g OGTT over 2 h Pre-op and at 6 months post-op	<60 mg/dL (3.3 mmol/L)	44% of SG
Raverdy, 2016 (24)	RYGB, *n*=957 (prospective unselected cohort)	75 g OGTT over 2 h, pre- and 1 and 5 years post surgery	<50 mg/dL (2.8 mmol/L) and plasma insulin >3 mU/L at 120 min−	0.5% at baseline, 9.1% and 7.9% at 12 months and 60 months

LAGB, laparoscopic adjustable gastric banding; OGTT, oral glucose tolerance test; RYGB, Roux-en-Y gastric bypass; SG, sleeve gastrectomy; T2DM, type 2 diabetes mellitus.

### Mixed meal test

Mixed meal tests (MMT) typically consist of a variable mix of carbohydrate, fat and protein administered most commonly as an orally ingested liquid with sampling for glucose up to 120–300 min. In a large unselected cohort, high rates of hypoglycaemia were seen, whilst even in asymptomatic people, hypoglycaemia is common (12). A meta-analysis of these studies is not possible as there is variability not only in terms of the stimulus but also the cut-offs for hypoglycaemia (Table 3). There is also limited correlation between induced hypoglycaemia and symptoms. False positives, side effects and lack of definition of meal composition and glycaemic cut-offs prevent routine clinical application.

**Table 3:** Summary table of studies of MMT in PBH.

Study	Population	Type of MMT when the test was done	Definition of hypoglycaemia	Prevalence of hypoglycaemia
Gasser, 2019 (42)	RYGB, *n* = 113, symptoms not stated	Carbohydrate rich MMT (201 kcal) Low-carbohydrate MMT (195 kcal) in PBH subset (*n* = 13) At 12 months post-op, 150 min	<54 mg/dL (3.0 mmol/L)	13.2% of RYGB with carbohydrate-rich MMT; none identified with PBH had hypo with low-carbohydrate MMT
Roslin, 2014 (31)	RYGB, *n* = 12 VSG, *n* = 12 DS, *n* = 12 Symptoms not stated	Mixed-meal in form of muffin containing 100 g glucose, post-op interval 9 months, 120 min	Not stated	Average 2 h glucose for RYGB= 85.3 ± 41 mg/dL (4.7 ± 2.3 mmol/L); VSG = 89.3 ± 39 mg/dL (5 ± 2.2 mmol/L); DS = 83.3 ± 23 mg/dL (4.6 ± 1.3 mmol/L)
Faro, 2018 (43)	Symptomatic RYGB, *n* = 5	MMT stimulus: 500 kcal, 20% protein, 20% fat and 60% carbohydrates), post-op interval 1.5–10 years, 120 min	Not stated	Nadir glucoses at 120 min of 45; 34; 52; 26; 43 mg/dL (respectively 2.5; 1.9; 2.9; 1.4; 2.4 mmol/L)
Goldfine, 2007 (9)	Symptomatic RYGB, *n* = 12 Asymptomatic RYGB, *n* = 9 Non-RYGB similar BMI pre-op, *n* = 5 Non RYGB similar BMI post-op, *n* = 10	Ensure, 9 g protein, 40 g carbohydrates, 6 g fat, 240 mL, post-op interval 0.8–3.8 in symptomatic group, 2–4 years in asymptomatic group, 120 min	<60 mg/dL (<3.33 mmol/L)	3/9 asymptomatic people developed asymptomatic hypoglycaemia
Honka, 2019 (44)	Symptomatic RYGB, *n* = 43 Asymptomatic RYGB, *n* = 52 Separate cohort Symptomatic RYGB, *n* = 31 Asymptomatic RYGB, *n* = 18	350 kcal MTT (Ensure Plus®, 51 g carbohydrates) Isovolemic 250 kcal liquid MTT (Ensure®, 40 g carbohydrates) post-op interval not stated Sampling duration not stated	Not stated	A nadir blood glucose cut-off of 53 mg/dL (2.9 mmol/L) produced the highest sum of sensitivity (65%) and specificity (88%) for detecting hypoglycaemia
Jacobsen, 2012 (45)	Eight people pre and 2 weeks after RYGB	200 mL liquid mixed meal test (Nutridrink, Nutricia, Netherlands) containing 300 kcal (1260 kJ) with 49% carbohydrate, 35% fat and 16% protein. Also compared to 25 and 50 g OGTT, 210 min	Not stated	No change pre- and post-operatively
Kefurt, 2015 (46)	Symptoms not stated, RYGB, *n* = 51	200 mL liquid (all in® COMPLETE Kaffee, Allin, Vienna) containing 7.2 g fat, 28.4 g carbohydrates and 16 g protein, post-op interval mean 7.2 years, 180 min	<55 mg/dL (3.1 mmol/L)	29%
Lee, 2022 (47)	Symptomatic SG, *n* = 12 Asymptomatic SG, *n* = 11 Symptomatic RYGB, *n* = 7 Asymptomatic RYGB, *n* = 13	8-ounce Ensure Plus drink (Abbott Laboratories; 350 kcal, 50 g carbohydrates, 13 g protein, and 11 g fat), post-op interval average 5 years, 240 min	<60 mg/dL (3.3 mmol/L)	SG 39%, RYGB 40%
Lobato, 2020 (12)	Symptomatic RYGB, *n* = 14 Asymptomatic RYGB, *n* = 9	Liquid Mixed Meal (Fresubin Energy Drink, 200 mL, 300 kcal (50E% carbohydrate, 15E% protein and 35E% fat); Fresenius Kabi Deutschland, Bad Homburg, Germany), post-op interval ~3–5 years, 120 min	<55 mg/dL (3.1 mmol/L)	50% symptomatic, 44.4% asymptomatic
Mulla, 2019 (15)	Symptomatic RYGB, *n* = 11 Asymptomatic RYGB, *n* = 7 Overweight non operated, *n* = 3 Obesity non operated, *n* = 4	(Ensure, 40 g carbohydrates, 9 g protein, 6 g fat, 240 mL; Abbott Laboratories, Abbott Park, IL) Post-op interval not stated, 120 min	Not defined	Nadir glucose (mmol/L) (RYGB symptomatic: 4.3 ± 0.2; RYGB asymptomatic: 3.9 ± 0.1; Overweight non operated: 5.5 ± 0.2; Obesity non operated: 5.3 ± 0.3
Patti, 2015 (48)	Symptomatic RYGB, *n* = 17 Asymptomatic RYGB, *n* = 6	(Ensure, 40 g carbohydrates, 9 g protein, 6 g fat, 240 mL) post-op interval ~3–5 years, 120 min	<60 mg/dL (3.33 mmol/L)	Hypos detected in 7/17 (41%) of the symptomatic group 0/9 of the asymptomatic group had hypos
Preskill, 2015 (49)	Symptomatic RYGB, *n* = 31 Asymptomatic RYGB, *n* = not reported	MMT containing carbohydrate, fat, and protein (in solid form), post-op interval not reported, 300 min	<55 mg/dL (<3.1 mmol/L)	2/31 of the symptomatic group Asymptomatic not reported
Raverdy, 2017 (50)	RYGB, *n* = 89 AGB, *n* = 20 Unselected prospective cohort study	MMT not specified, pre-surgery, 12 and 60 months post surgery, 180 min	Not specified	2 h post-prandial lower after RYGB than AGB
Salehi, 2010 (51)	Symptomatic RYGB, *n* = 20 with CBG <50 mg/dL Asymptomatic RYGB, *n* = 21	MMT 350 kcal not specified, post-op interval and sampling duration not reported	Not specified	Nadir glucose <50 mg/dL (<2.8 mmol/L) in 9/20 symptomatic and 5/21 asymptomatic
Smajis, 2016 (52)	Symptomatic RYGB (GB), *n* = 10 Symptomatic of hypoglycaemia but with no surgery (CON), *n* = 5	Solid MMT (one meal consisting of 40 g whole wheat bread, 15 g butter, 20 g honey and 100 g banana (total kcal: 360; 4 g protein, 13 g fat, 57 g carbohydrate), post-op interval not reported, 240 min	<55 mg/dL (3.1 mmol/L)	Asymptomatic hypoglycaemia detected in GB = 2/10 and CON = 2/5
Emous, 2019 (53)	RYGB, *n* = 44	200 mL liquid nutrition supplement (Abbott Ensure S Plus) containing 300 kcal, 12.5 g protein, 40.4 g carbohydrate (of which 13.8 g is sugars), 9.84 g fat and 154.9 g water MMT, 4 years post-op, 210 min	<60 mg/dL (3.3 mmol/L)	Hypos in 48%, all asymptomatic
Tharakan, 2017 (54)	Symptomatic RYGB, *n* = 18 Asymptomatic RYGB, *n* = 10 non-RYGB controls, *n* = 9	Ensure Plus food supplement (13.8 g of protein, 10.8 g of fat, 44.4 g of carbohydrates, 330 kcal, 220 mL, Abbott)	<3.0 mmol/Ll	Hypos in 7/18 symptomatic people
Johannson, 2010 (55)	BPD-DS, *n* = 10 Normal weight controls, *n* = 12	Mixed meal including hamburger, and jam sandwich with margarine and oat bread, 2400 kJ (570 kcal), consisting of: carbohydrates 68.2 g, fat 22.3 g, proteins 24.6 g and fibre 6.4 g. 2 years post-op, 180 min	Not prespecified	No reported hypoglycaemia

BPD-DS, biliopancreatic diversion with duodenal switch; DS, duodenal switch; (L)AGB, (laparoscopic) adjustable gastric banding; MMT, mixed meal test; RYGB, Roux-en-Y gastric bypass; SG, sleeve gastrectomy; VSG, vertical sleeve gastrectomy.

### Continuous glucose monitoring

Continuous glucose monitoring (CGM), whether using real-time systems such as the Dexcom G-series sensor system or the Medtronic Guardian system, or intermittently scanned systems such as the Abbott FreeStyle Libre system, allows continuous measurement of interstitial glucose concentrations via easily and painlessly inserted subcutaneous sensors. Such technologies are purchasable by people and are now prescribable in the NHS for diabetes mellitus monitoring, although they are not presently prescribable for people with PBH.

The current research, summarised in Table 4, suggests CGM has potential for detecting PBH. From CGM studies, PBH is related to elevated glycaemic variation (GV) utilising various metrics such as the percentage coefficient of variation (%CV), mean absolute glucose (MAG) and mean absolute glucose excursion (MAGE). CGM, by measuring day-to-day glucose concentrations in reaction to a particular person’s diet, may be more reflective of their experience of hypoglycaemia than a standardised dynamic test. However, most studies have used CGM systems which are now obsolete and collected the data for relatively short periods of time, limiting their interpretation.

**Table 4:** Summary table of studies of CGM in PBH.

Study	Population	Investigation	Definition of hypoglycaemia	Outcome
Rett, 2013 (56)	Symptomatic RYGB, *n* = 27	Dexcom seven^+^ CGM over 7 days	<70 mg/dL (<3.9 mmol/L)	Hypoglycaemia detected in 93%. Raised GV as measured by MAG.
Nielsen, 2015 (57)	Symptomatic RYGB, *n* = 12 Asymptomatic RYGB, *n* = 12	Not specified CGM over 6 days Both sets of people subjected to 5-h MMT, low-carbohydrate diet for 1 day, ordinary diet for rest of monitoring period	Not specified	CGM glucose levels overestimate plasma glucose during an MMT by 1.0 mmol/L. Raised GV as measured by MAGE during low carbohydrate diet lower than during ordinary diet.
Ritz, 2016 (58)	Symptomatic non-specified bariatric surgery, *n* = 142 Asymptomatic non-specified bariatric surgery, *n* = 25	Not specified CGM system	<60 mg/dL (<3.33 mmol/L) Cohort divided into those with hypos and those without	Hypo people had more time in hypo <60 mg/dL and GV. No difference in symptoms of hypos between people with hypos and those without. Sigstad dumping syndrome score higher in hypo people.
Halperin, 2011 (59)	Asymptomatic RYGB, *n* = 6 Symptomatic RYGB, *n* = 10	Medtronic Minimed iPro CGM for 72–120 h All people also subjected to MMT (Ensure, 9 g protein, 40 g carbohydrates, 6 g fat, 240 mL; Abbott)	<70 mg/dL (<3.9 mmol/L)	9/10 symptomatic people had hypos on CGM and 3/9 on MMT. 3/6 asymptomatic people had hypos on CGM and 3/5 on MMT. Sensitivity of CGM for clinically significant hypo was 90% and specificity 50% (33%, 40% for MMT).
Kefurt, 2015 (46)	Symptoms not stated RYGB, *n* = 51	Medtronic iPro-2 CGM over 5 days	<55 mg/dL (<3.1 mmol/L)	75% had hypo episodes with mean duration 71 min.
Capristo, 2018 (29)	Randomly selected RYGB, *n* = 25 Randomly selected SG, *n* = 25 (out of cohort of 120 people assigned 1:1 to RYGB vs SG)	Medtronic Enlite for up to 5 days	<55 mg/dL (<3.1 mmol/L)	No difference in average daily glycaemic values ≤3.1 mmol/L nor daily average number of hypo episodes between RYGB vs SG.
Lobato, 2020 (60)	Symptomatic RYGB, *n* = 13	Abbott FreeStyle Libre for 14 days, first 48 h excluded	<70 mg/dL (<3.9 mmol/L) with concurrent hypoglycaemic symptoms or <54 mg/dL (<3.0 mmol/L)	Significant hypoglycemia (time in range <54 mg/dL/<3.0 mmol/L ≥1%) in 8/13.
Ilesanmi, 2021 (13)	Asymptomatic RYGB, *n* = 10	Dexcom G4 or G6 CGM for up to 10 days Assessed before surgery, at 1 month, 1 year and 2 years after surgery MMT with Ensure Compact (13 g protein, 11.6 g fat, 36 g carbohydrates, 330 kcal, 137.5 mL; Abbott) before surgery, at 1 month, 1 year	<3.0 and <3.9 mmol/L	Significant hypoglycaemia (time in range <3.0 mmol/L >1%) in 5/10. GV similar between baseline pre-surgery and at 1 month, but increased at 1 year and 2 years after surgery. Prevalence of asymptomatic hypo in Roux-en-Y gastric bypass was 50%.

CGM, continuous glucose monitoring; GV, glycaemic variation; (L)AGB, (laparoscopic) adjustable gastric banding; MAG, mean absolute glucose; MAGE, mean absolute glucose excursion; MMT, mixed meal test; RYGB, Roux-en-Y gastric bypass; SG, sleeve gastrectomy.

### Considerations for our recommendations for diagnosis

We recommend a pragmatic definition of PBH in a patient after bariatric surgery as typical hypoglycaemic symptoms which are well recorded with biochemically confirmed hypoglycaemia <3.0 mmol/L (54 mg/dL) preferably utilising a venous plasma sample and an assay which is validated for low glucose concentrations, and resolution of symptoms with reversal of hypoglycaemia, thus fulfilling Whipple’s triad. Alternative causes of hypoglycaemia should be considered, investigated and excluded (Recommendations 1.1, 1.2, 1.3, Table 1).

Dynamic provocation tests are not recommended for the diagnosis of PBH (Recommendation 1.4, Table 1) for the following reasons: (1) OGTT is a non-physiological stimulus which provokes post-prandial hypoglycaemia in 5.5% of people with no diabetes as well as a high proportion of people postoperatively who may be otherwise well; (2) although MMT attempts to reproduce the mixture of carbohydrates, fat and proteins in a meal, these stimuli are not standardised, and it is arguable whether a liquid dietary supplement reflects a true mixed meal due to differences in gastric emptying and digestion. Cut-offs for hypoglycaemia on provocation testing are non-standardised ranging from 2.8 to 3.3 mmol/L (50–60 mg/dL). Given the often-delayed hypoglycaemia post-prandially, measurement post provocation may need to continue to 3 h if not 4 hours in people reporting particularly delayed clinical features of hypoglycaemia, increasing the cost of diagnosis.

We do not presently recommend CGM for diagnosis of PBH (Recommendation 1.5, Table 1) for the following reasons: (1) the accuracy of these devices in the hypoglycaemic range is insufficient (61); (2) it is not presently clear what the expected ‘normal’ response of glycaemia to bariatric surgery is (62); (3) CGM may be over-sensitive, detecting interstitial hypoglycaemia without apparent symptoms in people who have had bariatric surgery (13) – it is presently unknown what the clinical implication of this phenomenon is; (4) healthy people exhibit some hypoglycaemia <3.9 mmol/L (70 mg/dL) on CGM (63); and (5) defined and agreed-upon metrics for identifying PBH on CGM do not currently exist. We recommend that more research needs to be conducted to define the diagnostic performance and cost-effectiveness of CGM in the diagnosis of PBH (Recommendation 6.1, Table 1), and the relationship of low interstitial glucose readings from CGM to the symptoms of PBH (Recommendation 6.2, Table 1).

With regard to the use of capillary blood glucose measurements, many current CE-marked capillary blood glucose measuring systems still exhibit relatively large biases at hypoglycaemic levels in excess of ISO 15197 standards which can make diagnosis of hypoglycaemia problematic (64). Notwithstanding this caveat and noting that it may be practically difficult to capture a venous plasma glucose at the time of hypoglycaemia, we pragmatically recommend that capillary blood glucose readings <3.0 mmol/L (54 mg/dL) can be acceptable if they are taken using a testing system which conforms to ISO 15197 standards, if there are multiple confirmatory readings, and if the history is strongly consistent with PBH (Recommendation 1.6, Table 1). In this spirit, in people who have suspected PBH but no biochemically proven hypoglycaemia, if capture of a venous plasma glucose is not practical or possible, then issuing a capillary blood glucose meter is a pragmatic strategy for diagnosis. We also recommend that more research needs to be conducted into the diagnostic performance and cost-effectiveness of capillary blood glucose measurements for the diagnosis of PBH (Recommendation 6.1, Table 1).

A suggested framework for diagnosis is presented in Fig. 1.

## Treatment options for PBH

### Dietary and behavioural modification

Previously published guidelines and position statements typically contain minimal dietary guidance despite acknowledging it as the mainstay of treatment (19, 65). Small published series including conference abstracts suggest that >90% of people respond to dietary interventions whilst an unpublished systematic review suggests a more modest response to dietary intervention (66). We recommend that people diagnosed with PBH should undergo evaluation by a registered dietitian, preferably with a specialist interest in bariatric surgery, with the aim of providing individualised guidance on a healthy and sustainable post-bariatric eating strategy (Recommendation 2.1, Table 1). Noting the high prevalence of malnutrition after bariatric surgery, people should also be screened and treated for nutritional deficiencies following accepted guidance such as the British Obesity and Metabolic Surgery Society’s guidance (67) (Recommendation 2.2, Table 1).

Although the major focus of dietary intervention is the restriction of carbohydrates, the role of protein adequacy, fats, meal timing and spacing, caffeine, alcohol and eating behaviours have been considered in detail as part of a pragmatic review by Suhl *et al.*, including a 10-point nutrition plan with emphasis on the importance of detailed and personalised assessment (68). The evidence synthesis below and associated summary of the evidence base are best employed by dietitians with a particular interest and expertise in bariatric surgery though is also intended to be of use in less expert settings that may be resource-limited. The available data identified from this scoping review and summarised below is reliant on a combination of small experimental feeding studies including uncontrolled and crossover design studies as well as retrospective uncontrolled studies of interventions delivered in a real-world setting.

### Carbohydrate restriction

Controlled portions of carbohydrates, emphasising low glycaemic index (GI) carbohydrates and avoiding rapidly absorbed carbohydrates, are central to dietary treatment (68). This should go hand-in-hand with the positive reinforcement of a healthy post-bariatric eating strategy. Unfortunately, adherence to this strategy is often inadequate in both those with PBH and controls, with both groups exceeding recommendations for added sugar.

Much of the data relating to the effect of carbohydrate restriction is from small uncontrolled feeding studies (69, 70, 71). For example, a test meal with a carbohydrate load of 30 g did not result in hypoglycaemia in 14 people with PBH (70). A retrospective study of 41 people with PBH that included patient interviews as well as medical records review found that guidance to eat up to 6 small meals a day with <30 g of carbohydrates/meal was associated with a reduction in hypoglycaemic frequency, nocturnal symptoms, increased nadir glucose as well as improvements to quality of life (72).

Therefore, we advise starting with dietary advice including small and frequent meals, reduced carbohydrate consumption to <30 g/meal and an emphasis on choosing low GI over high GI carbohydrates. Consideration may be given to lower targets (e.g. 25 g/meal, 20 g/meal and so on) under supervision if the initial 30 g/meal target is not effective, but this needs to be supervised (Recommendation 2.3, Table 1).

### Modification of carbohydrate types and glycaemic index of carbohydrates

Modifying carbohydrate type can also make an impact. A small, randomised crossover study demonstrated that fructose-containing meals had more modest effects on insulin and glucose variability than glucose-containing meals in people with PBH (73). However, increased fructose intake cannot be fully recommended given its potential role in fostering metabolic dysfunction-associated fatty liver disease and increasing cardiovascular risk (74).

Whilst cornstarch supplementation has been proposed, this scoping review only identified one study in which cornstarch was used in two people unresponsive to prior dietary interventions, resulting in an improvement in hypoglycaemic frequency on CGM (75).

Various food additives such as pectin, guar gum and glucomannan have been studied as methods to slow gastric emptying and nutrient absorption mainly in the context of late dumping syndrome, with some evidence for efficacy in reducing post-prandial hypoglycaemia (76, 77, 78, 79, 80, 81, 82, 83, 84, 85). However, no published evidence for the applicability of these additives to PBH exists, and many people are unable to tolerate the additives as they change the texture and palatability of foods.

At present, we do not recommend these dietary manoeuvres (Recommendation 2.4, Table 1).

### The use of CGM for the management of hypoglycaemia

CGM may have a role in educating people, reinforcing and documenting the relationship of dietary changes to the amelioration of PBH (60, 86, 87), therefore improving adherence to dietary advice. CGM technology now enables users to set alarms to alert them to incipient hypoglycaemia, which may allow them to take preventive action, especially where there is hypoglycaemic unawareness. A small study suggested that unblinded CGM is associated with improvements in time in range (3.9–10 mmol/L, 70–180 mg/dl) compared to blinded CGM, with reductions both in time below range (<3.9 mmol/L, 70 mg/dl) and above range (>10 mmol/L, 180 mg/dl) (88). In summary, there is early evidence that CGM may be helpful in improving the management of PBH (Recommendation 2.5, Table 1).

At present, NICE recommendations do not include the use of CGM for this purpose, thus making access to the technology difficult for people who cannot otherwise afford to acquire the sensors. Stronger evidence for the efficacy and cost-effectiveness of this approach needs to be developed to enable the funding of this technology (Recommendation 6.3, Table 1).

### Emergent treatment of hypoglycaemia

Hypoglycaemia treatment should follow the general principles of management as in diabetes with the additional note that sucrose should be avoided for treatment in people treated with acarbose (68, 89). Some units recommend a strategy of staged treatment of hypoglycaemia to avoid precipitating more PBH. This involves taking small quantities of fast-acting carbohydrates (e.g. 10–15 g of dextrose) to obviate re-precipitation of PBH, with checks of blood glucose every 15 min to decide on further dosing. Once hypoglycaemia is resolved, a low glycaemic index carbohydrate containing snack may be considered (Recommendations 2.6, 2.7, Table 1). However, more research is required into the optimal method/strategy to treat hypoglycaemia in PBH (Recommendation 6.4, Table 1).

### Management of exercise

Although exercise can precipitate symptoms of PBH in some people, it is generally considered safe at least 3 months after surgery, with exercise-associated hypoglycaemia being uncommon. A small study of nine people suggested that moderate or intense exercise whilst fasting in people reporting PBH was not associated with hypoglycaemia (90).

Practically, people with PBH should be advised to exercise regularly to assist with weight maintenance after bariatric surgery, but to exercise at times when there is a low risk of PBH (e.g. when fasting, or at least 3 hours after eating). They should avoid carbohydrate loading prior to exercise, measure their glucose levels if they feel unwell, treat any emergent hypoglycaemia during exercise as above and consider a low GI carbohydrate-containing snack after exercise (Recommendation 2.8, Table 1).

### Caffeine

Some guidance suggests that a trial of reducing caffeine intake can be helpful (68). Theoretically, caffeine can exacerbate the initial rapid rise of glucose and insulin after eating. However, there is currently no specific evidence to directly demonstrate that reducing caffeine intake is helpful for PBH.

### Reduction of alcohol consumption

Alcohol consumption reduces glucose levels, at least in part mediated by a reduction in hepatic gluconeogenesis. The effect after bariatric surgery is likely to be dose dependent with modest amounts of alcohol consumed without food having similar effects post-surgery compared to pre-surgery, despite a marked effect of surgery on the blood alcohol concentration post ingestion (91). Most alcoholic drinks contain varying levels of non-fermented high GI carbohydrates, and as such, even without the effect of alcohol, these drinks can trigger PBH. Lastly, there is a higher risk of alcohol use disorder after bariatric surgery (92). Therefore, avoiding alcohol is good practice in people with PBH and after bariatric surgery in general (Recommendation 2.9, Table 1).

### High dietary protein intake

Protein adequacy is important post-bariatric surgery as protein malnutrition is a serious complication. Current guidance for nutritional management after bariatric surgery emphasises a role for enhanced protein intake, minimally 60 g/day, although higher individualised targets may be required (Recommendation 2.10, Table 1) (93). Protein consumption is also likely to be relevant in the management of PBH given the role of protein in GLP-1, glucagon and insulin secretion and in altering gastric emptying/intestinal motility when consumed at the same time as carbohydrate. In ten people with post RYGB hypoglycaemia, a randomised cross-over experimental meal study examined the differences between a conventionally recommended (CR) and a carbohydrate-reduced high-protein diet (CRHP) and found less glycaemic variability in the CRHP diet with no hypoglycaemia following the CRHP test meals. The CRHP was 30/30/40 by percentage energy supplied by, respectively, carbohydrate/protein/fat, whilst the CR diet was 55/15/30. About 15% of total energy expenditure was given as a breakfast test meal and 20% as a lunch test meal (94). The importance of protein adequacy is supported by a study of 17 people with prior RYGB of whom half were symptomatic for PBH. The study incorporated CGM, food and symptom diary and demonstrated that meals prior to hypoglycaemia were more likely to be lower in protein or higher in sugar (95).

### Modification of meal volume and fluid intake

Although not specifically studied in people with PBH, feeding studies in people with RYGB demonstrate a physiological basis for the reduction in meal volume as well as a trial of supine posture in people with PBH (96). In a carefully controlled feeding study of people pre and post RYGB, solid rather than liquid meals were associated with a lower frequency of hypoglycaemia and a reduction in peak GLP-1 response to feeding. The effect of small, regular rather than large, single meals was less certain, though as anticipated, small regular meals were associated with a reduction in insulin response to feeding (97). This study provides clear evidence of the importance of solid rather than liquid meals as well as smaller volume regular meals. This is the basis for the commonly delivered guidance to people with PBH to avoid fluids with meals (68). Ideally, people should avoid drinking in the 30 min prior to and continuing for 30 min after each meal, and minimally they should avoid drinking with the meal and for at least 30 min after each meal (Recommendation 2.11, Table 1).

### Gastrostomy feeding tubes

Gastrostomy feeding tube placement into the remnant stomach has been proposed as a possible treatment option for people with resistant PBH post RYGB by ASMBS, and the practical experience of one of the authors (RA) has shown that this may be a useful option in severe cases of PBH. However, available data is limited to small case series, including a study in which only four of the six people identified as having treatment-resistant post-bariatric surgery hypoglycaemia had a nadir glucose <2.8 mmol/L on OGTT, an experimental study in which six people awaiting RYGB reversal were shown to have a much less marked GLP-1, insulin and hypoglycaemic response to a liquid MMT given via tube compared to oral feeding, and a more recent series detailing outcomes of treatment response and failure in those with PBH (98). Nevertheless, we recommend that this may be an option in severe PBH which does not respond to dietary nor pharmacological treatment (Recommendation 2.12, Table 1).

## Pharmacotherapy

### Glucosidase inhibitors

Acarbose is a competitive inhibitor of pancreatic α-amylase and intestinal brush border α-glucosidases, causing a delayed hydrolysis of ingested polysaccharides, oligosaccharides and disaccharides to monosaccharides. Acarbose, administered to people with symptomatic PBH, results in blunting of the postprandial rise in plasma glucose, which in turn reduces insulin secretion during a mixed meal study. The effect of acarbose on postprandial levels of GLP-1 is controversial, with some studies showing no change and others reporting a reduction in the peak GLP-1. Under real-life conditions using CGM, acarbose significantly reduces time in hyperglycaemia and time in hypoglycaemia (99). In the retrospective series of de Heide *et al.*, 107 out of 120 people with PBH were treated with acarbose at a median dose of 150 mg daily, of whom 21% were reported to have complete resolution of all hypo events, 35% had 50–100% resolution, 20% had 20–50% resolution and 24% had 0–20% resolution (minimal or no resolution). From the patient's perspective, the majority (61%) of people treated with acarbose reported either no hypos, almost no hypos, or an acceptable frequency of hypos. Although effective in treating PBH, there are gastrointestinal side effects such as abdominal pain, flatulence, bloating, and diarrhoea, which lead to long-term attrition of adherence (100). Despite these limitations, acarbose has been used to treat people with PBH for as long as 120 months (100, 101). Simethicone may also be considered as a means of easing the flatulence from acarbose treatment (102).

In summary (Table 5), there is sufficient clinical experience with acarbose to suggest that it has moderate efficacy for PBH. Considering its relatively low cost and side effect profile, we suggest that it is considered as the first-line pharmacotherapy for PBH (Recommendation 3.1, Table 1).

**Table 5:** Summary table of studies of acarbose in PBH.

Study	Population	Intervention	Comparator	Outcome
Valderas, 2012 (103)	RYGB with symptomatic PBH, *n* = 8	Acarbose 100 mg single dose 15 min before MMT	No acarbose before MMT (unblinded)	No episode of hypoglycaemia <50 mg/dL (2.8 mmol/L) during MMT with acarbose. Reduction in insulin AUC and reduction in the peak GLP-1 during MMT.
Cadegiani, 2016 (102)	RYGB with symptomatic early and late dumping syndrome, refractory to dietary intervention, *n* = 25	Acarbose 50 mg, four to five times a day for 6 months, with simethicone 120 mg twice a day for people with flatulence (to attenuate side effects of acarbose)	No comparator	85.8% reduction in episodes of early dumping and 95.7% reduction in episodes of late dumping (PBH) per week. Improvement in symptoms of early dumping as assessed by Sigstad score.
Frankhouser, 2013 (101)	RYGB with symptomatic PBH, *n* = 5	Acarbose, for between 5 and 48 months	No comparator	Symptomatic hypoglycaemia resolved in four people. One patient discontinued due to rash.
Ohrstrom, 2019 (99)	RYGB; symptomatic PBH, *n* = 11	Acarbose 50 mg four to six times a day for 7 days	Compared to no-treatment baseline (part of cross-over study with sitagliptin, verapamil, pasireotide, liraglutide)	Reduced peak and increased nadir glucose during MMT comparing after treatment to pre-treatment. Reduced insulin and C-peptide level during MMT. No difference in peak GLP-1 during MMT. Reduced time in hyperglycemia and glycaemic variability during CGM after treatment compared to pre-treatment. No significant difference in time in hypoglycaemia (<3.9 or 3.0 mmol/L).
de Heide, 2023 (100)	*n* = 120 cases of PBH, of whom 82/120 had RYGB, 34/120 had SAGB, 3/120 had SG and 1 SADI	Acarbose, median dose 150 mg daily, in 107 people, for up to 120 months	No specific comparator (case series)	21% had 100% reduction of hypos, 35% had 50–100% reduction, 20% had 20–50% reduction, 24% had 0–20% reduction.

MMT, mixed meal test; RYGB, Roux-en-Y gastric bypass; SG, sleeve gastrectomy; SADI, single-anastomosis duodenal-ileal bypass; SAGB, single-anastomosis gastric bypass, also known as one-anastomosis gastric bypass.

### GLP-1 analogues

In small case series of people with PBH, treatment with the GLP-1 analogue liraglutide improved symptoms of hypoglycaemia, fasting and post-prandial glucose concentrations, lowered insulin levels during an OGTT and in one case reduced time in hypoglycaemia on CGM. In the case series reported by de Heide and co-workers, 13 people with PBH were treated with liraglutide or semaglutide for up to 60 months with a complete response (100% reduction in hypo episodes) in 1 out of 13, 50–100% reduction in hypo episodes in 8 out of 13, 20–50% reduction in hypo episodes in 1 out of 13 and 0–20% reduction in hypo episodes in 2 out of 13, suggesting some partial efficacy (100). The mechanism by which the GLP-1 agonist ameliorated hypoglycaemia in these case series is unclear. In a short-term study in people who had undergone RYGB (albeit without histories of PBH), exenatide was not shown to influence counter-regulatory hormone secretion (glucagon, catecholamines, growth hormone, cortisol) during a hypoglycaemic hyperinsulinemic clamp, suggesting that if there is any effect of GLP-1 analogues, it is not mediated through that mechanism (104).

Contrary to the abovementioned studies, a cross-over study examining the effect of 3 weeks’ treatment with liraglutide in 11 women with documented PBH did not show any evidence of significant change in time in hypoglycaemia, although glucose variation was reduced on CGM.

In summary (Table 6), insufficient evidence currently exists to recommend the use of GLP-1 analogues in PBH (Recommendation 3.6, Table 1).

**Table 6:** Summary table of studies of GLP-1 analogues in PBH.

Study	Population	Intervention	Comparator	Outcome
Abrahamsson, 2013 (105)	Symptomatic RYGB, *n* = 5	Liraglutide 1.2 mg once a day	none	No symptoms in people with treatment. CGM record in one patient showed significant hypoglycaemic dips prior to treatment and no time in hypoglycaemia with treatment.
Stier, 2015 (106)	Symptomatic RYGB, *n* = 7	Liraglutide 0.6 mg and 1.2 mg once a day	none	Improvement in glycaemic symptoms reported with resolution of symptoms in 6/7. Fasting and OGTT insulin levels reported reduced.
Ohrstrom, 2019 (99)	Symptomatic RYGB, *n* = 11	Liraglutide 1.2 mg once a day for 3 weeks	Compared to no-treatment baseline (part of cross-over study with sitagliptin, verapamil, pasireotide, acarbose)	No significant effect on nadir glucose during MMT. No difference in time in hypoglycaemia; reduced glycaemic variation during CGM.
de Heide, 2023 (100)	*n* = 120 cases of PBH, of whom 82/120 had RYGB, 34/120 had SAGB, 3/120 had SG and 1 had SADI	Liraglutide (median dose 1.8 mg/day) or semaglutide (median dose 1 mg/week), in 13 people for up to 60 months	No specific comparator (case series)	1/13 had 100% reduction of hypos, 8/13 had 50–100% reduction, 1/13 had 20–50% reduction, 2/13 had 0–20% reduction.

CGM, continuous glucose monitoring; MMT, mixed meal test; OGTT, oral glucose tolerance test; RYGB, Roux-en-Y gastric bypass; SG, sleeve gastrectomy; SADI, single-anastomosis duodenal-ileal bypass; SAGB, single-anastomosis gastric bypass, also known as one-anastomosis gastric bypass.

### GLP-1 receptor antagonism

As discussed earlier, a leading hypothesis for the pathogenesis of PBH involves the excessive secretion of GLP-1 by L-cells in reaction to the ingestion and absorption of nutrients, excessive hyperinsulinaemia and consequent ‘overshoot’ correction of blood glucose. Blockade of the GLP-1 receptor with the antagonist exendin 9–39 (also known by its recommended international non-proprietary name avexitide) leads to the correction of hypoglycaemia and reduced hyperinsulinaemia in response to an MMT or OGTT and reduced hypoglycaemic symptoms in people with PBH (10, 107, 108, 109). A phase II placebo-controlled crossover trial of subcutaneous avexitide at two doses (30 mg twice a day and 60 mg once a day) over 28 days in 18 people with PBH showed improvements in nadir glucose and peak insulin during an OGTT, reduction in hypoglycaemia without relevant increases in hyperglycaemia on CGM, and improvements in rates of hypoglycaemia defined as self-measured blood glucose (SMBG) <70 mg/dL (3.9 mmol/L), <54 mg/dL (3.0 mmol/L) and severe hypoglycaemic events with altered mental and/or physical function requiring assistance (110).

In summary, avexitide (exendin 9–39) appears to be effective in treating PBH (Table 7) but is not currently available outside of phase III clinical trials, as of writing. Referral for these trials, if available, may be a reasonable step for those who are willing to participate (Recommendation 3.5, Table 1).

**Table 7:** Summary table of studies of GLP-1 receptor antagonism in PBH.

Study	Population	Intervention	Comparator	Outcome
Salehi, 2014 (10)	Symptomatic RYGB (H-GB), *n*=9 Asymptomatic RYGB (A-GB), *n* = 7 Healthy controls, *n* = 8	Exendin 9–39 IV infusion	Saline infusion	Exendin 9–39 corrected post-prandial hypoglycaemia in all H-GB group.
Craig, 2017 (107)	Symptomatic RYGB, *n* = 10	Exendin 9–39 IV infusion in cross-over study	Placebo	During 75 g OGTT, exendin 9–39 reduced peak and increased nadir glucose (preventing development of hypoglycaemia).
Craig, 2018 (108)	Symptomatic RYGB, *n* = 9	Ascending SC dose of exendin 9–39	None	During 75 g OGTT, exendin 9–39 increased nadir glucose by and decreased peak insulin.
Tan, 2020 (109)	Symptomatic RYGB, *n* = 19 for lyophilised and *n* = 5 for liquid formulation	Ascending SC doses of exendin 9–39 (avexitide) in lyophilised and liquid formulations	None	During 75 g OGTT, avexitide improved glucose nadir, insulin peak and symptom scores in a dose-dependent manner. Doses ≥20 mg twice a day obviated need for rescue glucose treatment initiated at glucose <2.8 mmol/L.
Craig, 2021 (110)	Symptomatic RYGB, all women and diet-refractory, *n* = 18	Avexitide 30 mg SC BD or 60 mg SC OD for up to 2 weeks each in cross-over study	Placebo	In mixed-meal testing, avexitide increased nadir glucose by 21–26% and reduced peak insulin by 21–23%. Significant reductions in frequency of hypoglycaemic episodes.

BD, twice a day; IV, intravenous; OD, once a day; OGTT, oral glucose tolerance test; RYGB, Roux-en-Y gastric bypass; SC, subcutaneous.

## Glucagon and glucagon analogues

RYGB is associated with a reduced counter-regulatory response to post-prandial hypoglycaemia, including glucagon secretion (111, 112). There is some evidence that PBH and hypoglycaemia during mixed meal testing and during continuous glucose monitoring are associated with alterations in glucagon secretion (12, 13). Glucagon is currently licenced for the treatment of severe hypoglycaemia in people with diabetes, and in the UK, it exists as a powder and solvent formulation (GlucaGen®, Novo Nordisk) or a stabilised liquid formulation (Ogluo®, Tetris Pharma/Xeris). Dasiglucagon is a glucagon analogue in a liquid formulation (Zegalogue®, Novo Nordisk) which is currently licenced in the USA but not in the UK.

In a case study, Halperin *et al.* described the use of an IV glucagon infusion in one patient with PBH after RYGB and demonstrated that this eliminated the hypoglycaemia experienced after mixed meal testing, albeit with much-enhanced insulin secretion in response to eating (113). In exploratory clinical studies, Mulla *et al.* utilised liquid glucagon delivered via a subcutaneous infusion pump coupled with CGM and a predictive algorithm for hypoglycaemic episodes in 12 people with PBH. In comparison to a vehicle control, the glucagon delivery system, utilising bolus doses of 150–300 µg, was able to prevent post-prandial hypoglycaemia after mixed meal testing (112). However, as of writing, this pump system is not presently generally available in the clinical setting. Dasiglucagon has been studied in a cross-over trial: as a single dose of 80 or 200 µg prior to an MMT stimulus, it has been shown to be effective in increasing nadir glucose and reducing time in hypoglycaemia <3.9 mmol/L.

In summary, glucagon can be used to treat hypoglycaemia (Table 8) and may be considered as a rescue therapy in cases of frequent and severe PBH (Recommendation 3.4, Table 1).

**Table 8:** Summary table of studies of glucagon or glucagon analogues in PBH.

Study	Population	Intervention	Comparator	Outcome
Halperin, 2010 (113)	Symptomatic RYGB, *n* = 1	Glucagon IV infusion in cross-over study	Vehicle	During MMT, glucagon eliminated the post-prandial hypoglycaemia evident with vehicle infusion.
Mulla, 2020 (112)	Symptomatic RYGB, *n* = 12	Automated glucagon SC boluses of 150 and 300 µg in cross-over study	Vehicle	During MMT, the algorithm predicted hypoglycaemia and administered glucagon boluses, obviating the need for rescue glucose treatment, and increasing nadir post-prandial plasma glucose in comparison to vehicle.
Nielsen, 2022 (114)	RYGB with hypoglycaemia on CGM, *n* = 10	Single dose of 80 µg or 200 µg dasiglucagon in three-way cross-over trial	Placebo	Increased nadir glucose and reduced time in hypoglycaemia <3.9 mmol/L during MMT compared to placebo, for both doses of dasiglucagon.

CGM, continuous glucose monitoring; IV, intravenous; MMT, mixed meal test; RYGB, Roux-en-Y gastric bypass; SC, subcutaneous.

### Somatostatin analogues

Somatostatin analogues such as octreotide, lanreotide and pasireotide have been used in individuals with ‘dumping syndrome’ following upper gastrointestinal surgery, such as gastrectomy and peptic ulcer surgery, for many years. They have been found to be effective for both for early and late dumping syndromes, with late dumping syndrome being the analogous condition to PBH in individuals undergoing upper GI surgery such as oesophagectomy or gastrectomy (115, 116, 117, 118). These are thought to work by activating somatostatin receptors (particularly subtypes 2 and 5) and inhibiting the release of various gut and metabolic hormones such as gastrin, pancreatic polypeptide, glucagon, neurotensin, secretin, GLP-1, and insulin, all of which have been implicated in the pathophysiology of dumping syndrome. There are relative differences in activity between the presently available somatostatin analogues octreotide, lanreotide and pasireotide. Both octreotide and lanreotide exhibit greater activity at subtype 2 than subtype 5, whereas pasireotide activates both subtypes 2 and 5. The more marked hyperglycaemic effect from pasireotide relative to octreotide in non-bariatric surgery individuals is due to the relative ratio of subtype 5:2 activation. Both pasireotide and octreotide are able to suppress insulin secretion, but pasireotide is less able to suppress glucagon secretion than octreotide, explained by the fact that inhibition of insulin secretion in human pancreatic islets is mediated by subtypes 2 and 5, while inhibition of glucagon secretion is mainly via subtype 2 (119). The long-term safety of somatostatin analogues is established in their widespread use for the treatment of acromegaly, neuroendocrine tumours and other indications, with the most common side effects being steatorrhoea due to relative pancreatic exocrine deficiency and an increase in the risk of gallstone development.

With regard to PBH (Table 9), short-acting SC octreotide and pasireotide (99, 120) appear to be effective at preventing hypoglycaemia following OGTT or MMT. IM long-acting pasireotide is also effective, although SC pasireotide three times a day may be more effective than long-acting IM pasireotide (117). With regard to lanreotide, there is no published experience on this medication for PBH, although the experience with this analogue in dumping syndrome suggested no difference in late dumping symptom scores (118).

**Table 9:** Summary table of studies of somatostatin analogues in late dumping syndrome or PBH.

Study	Population	Intervention	Comparator	Outcome
Arts, 2009 (116)	Upper GI surgery including partial gastrectomy, RYGB, Nissen fundoplication, partial oesophagectomy; post-operative hypoglycaemia (<3.3 mmol/L) during OGTT, *n* = 30 *n* = 10 had had RYGB	SC Octreotide 50 µg three times a day for 3 days (cross-over study)	IM Octreotide LAR 20 mg monthly for 3 months	SC octreotide improved nadir glucose during OGTT although 7/30 still experienced significant hypoglycaemia. IM octreotide LAR (3 months’ treatment) improved nadir glucose during OGTT although 11/30 still experienced significant hypoglycaemia. Improvements in late dumping severity scores with both SC octreotide and IM octreotide LAR.
Tack, 2018 (117)	Upper GI surgery including RYGB, gastrectomy, oesophagectomy; symptomatic hypoglycaemia (late dumping symptoms), *n* = 43 for SC dose escalation phase (38 with RYGB). Only *n* = 33 (28 with RYGB) went on to IM phase	3-month dose escalation phase with SC pasireotide (50−200 µg three times a day) followed by 3-month IM phase (10 or 20 mg monthly), and optional 6-month extension phase with IM pasireotide	None (single-arm trial)	60.5% of people did not have hypoglycaemia at end of SC phase. 36.4% of people did not have hypoglycaemia at end of IM phase.
Wauters, 2019 (118)	Upper GI surgery: gastrectomy/bypass, oesophagectomy, non-resective oesophageal surgery; meeting criteria for early dumping syndrome, or late dumping syndrome (hypoglycaemia <60 mg/dL or 3.3 mmol/L) during OGTT, or history of hypoglycaemia <60 mg/dL or 3.3 mmol/L; *n* = 24 (2 arms of 12)	SC lanreotide Autogel 90 mg monthly over 3 months Double-blind, randomised, placebo-controlled crossover study where all people underwent 3 months of lanreotide and 3 months of placebo	Saline injection monthly over 3 months	Lanreotide improved early dumping symptom score but not late dumping symptom score (hypoglycaemia).
Whyte, 2010 (121)	Symptomatic RYGB, *n* = 4	Single dose of SC octreotide 100 µg	No treatment	Symptomatic hypoglycaemia during extended OGTT said to have been prevented by pre-treatment with octreotide.
de Heide, 2014 (120)	Symptomatic RYGB, *n* = 1	Single dose of SC octreotide 100 µg	Single dose of SC pasireotide 300 µg	Octreotide given prior to MMT resulted in hypoglycaemia (2.0 mmol/L) during MMT whereas pasireotide did not (3.5 mmol/L). SC Pasireotide 300 µg twice a day resulted in amelioration of hypoglycaemic episodes.
Ohrstrom, 2019 (99)	Symptomatic RYGB, *n* = 11	Single dose of SC pasireotide 300 µg prior to MMT	Compared to no-treatment baseline (part of cross-over study with sitagliptin, verapamil, liraglutide, acarbose)	Significant increases in nadir glucose during MMT.

CGM, continuous glucose monitoring; IM, intramuscular; IV, intravenous; LAR, long-acting release; MMT, mixed meal test; OGTT, oral glucose tolerance test; RYGB, Roux-en-Y gastric bypass; SC, subcutaneous.

In summary, there is evidence to suggest that short-acting SC octreotide and pasireotide can prevent PBH when given three times a day with meals (Recommendation 3.2, Table 1). IM pasireotide LAR is also effective (Recommendation 3.2, Table 1). However, less evidence for efficacy exists for other long-acting preparations, and these are not presently recommended (Recommendation 3.6, Table 1).

### SGLT inhibitors

In humans, the sodium–glucose linked transporters SGLT-1 and SGLT-2 play an important role in sodium and glucose transport across the basal brush membrane of gut and kidney cells. SGLT-1 is expressed in the enterocyte and in enteroendocrine cells (L cells and K cells) which, respectively, produce the incretins GLP-1 and GIP. SGLT-1 is responsible for glucose absorption in the small intestine and for the reabsorption of approximately 10% of the filtered glucose load in segment 3 of the renal proximal tubule. SGLT-2 is primarily expressed in the kidney and is responsible for the reabsorption of approximately 90% of the filtered glucose load in the proximal tubule segments 1 and 2.

In addition to SGLT-1 receptors, GLUT-2 receptors are essential for the transport of glucose across the basolateral membrane of the small intestine. After RYGB, there is upregulation of SGLT-1 and GLUT-2 expression (122). As SGLT-1 is present on the enteroendocrine cells, it is plausible that intestinal inhibition of SGLT-1 may reduce the exaggerated incretin rise seen after surgery. The consequence of this could be a lower peak glucose and theoretically reduced risk of subsequent hypoglycaemia.

Canagliflozin competitively inhibits SGLT-1 and SGLT-2 with a higher selectivity for SGLT-2 (123). Canagliflozin, when given to individuals who had undergone RYGB, led to a delay in glucose absorption and reduced peak glucose in response to a 50 g OGTT. There was no significant reduction in nadir glucose, albeit there was no evidence of PBH in this group. This was accompanied by a reduction in peak GLP-1 concentrations and later glucagon levels (124). In a pilot study involving 21 people with PBH after RYGB, pre-treatment with canagliflozin 300 mg reduced peak glucose and increased nadir glucose in response to a 100 g OGTT (125). In a case report, one patient with PBH was given canagliflozin 100 mg once a day for 9 months, with symptomatic improvement and a reduction in hypoglycaemia documented on intermittently scanned glucose monitoring and continuous glucose monitoring (126). There are formal clinical trials in progress, evaluating the effect of canagliflozin in PBH (127). Sotagliflozin similarly inhibits SGLT-1 and SGLT-2 with a higher selectivity for the latter, but at present, there are no recorded studies or trials of this medication for PBH.

In one trial conducted on a small number of people with symptomatic PBH, empagliflozin (a selective SGLT-2 inhibitor) reduced peak postprandial glucose, reduced insulin levels and reduced episodes of hypoglycaemia during an MMT (16). Subsequently, a randomised double-blind controlled trial of empagliflozin in 22 people, given 25 mg daily (or placebo) for 20 days, showed that post-prandial glucose excursions were reduced, but the time below range (<3.0 mmol/L) during CGM was no different between the placebo and treatment groups (128).

In summary, there is some anecdotal evidence to support the use of canagliflozin but no evidence for the use of empagliflozin in PBH (Table 10). Overall, there is presently insufficient evidence to recommend the routine clinical use of SGLT inhibitors for PBH (Recommendation 3.6, Table 1).

**Table 10:** Summary table of studies of SGLT inhibitors in PBH.

Study	Population	Intervention	Comparator	Outcome
Abouglila, 2017 (126)	Symptomatic RYGB, *n* = 1	Canagliflozin 100 mg a day for 9 months	none	Symptomatic improvement and a reduction in hypoglycaemia documented on glucose monitoring.
Ciudin, 2021 (125)	RYGB, symptomatic in 83%, *n* = 21	Canagliflozin 300 mg single dose prior to 100 g OGTT	No-treatment baseline 100 g OGTT	Significant reduction in peak and nadir glucose during OGTT. 20/21 people had nadir glucose <50 mg/dL (2.8 mmol/L) at baseline, 2/21 after canagliflozin.
Ferreira, 2023 (128)	RYGB, *n* = 22	Empagliflozin 25 mg daily for 20 days	Placebo	No difference in quantity and quality of symptoms, reduced glucose excursion after meals, no difference in time <3.0 mmol/L on CGM.
Hepprich, 2020 (16)	Symptomatic RYGB, *n* = 12	Empagliflozin 10 mg prior to MMT	Anakinra (IL-1beta antagonist) Placebo	Reduced hypoglycaemia episodes during MMT following empagliflozin compared to placebo.
Martinussen, 2020 (124)	Asymptomatic RYGB, *n* = 10	Canagliflozin 600 mg single dose prior to 50 g OGTT	Placebo	Canagliflozin led to a delay in glucose absorption and reduced peak glucose but not nadir glucose.

MMT, mixed meal test; OGTT, oral glucose tolerance test; RYGB, Roux-en-Y gastric bypass.

### Other treatments

Diazoxide is a potassium channel activator, leading to hyperpolarization and inhibition of beta-cell secretion of insulin. It has been described as a treatment for PBH in case studies (129, 130, 131). In the case series of de Heide *et al.*, 14 people were treated with diazoxide: 3/14 had 100% reduction in hypos, 4/14 had 50–100% reduction, 3/14 had 20–50% reduction in hypos and 5/14 had 0–20% reduction in hypos (100). Therefore, diazoxide is recommended as a possible third-line treatment (Recommendation 3.3, Table 1). However, tolerability of diazoxide is limited by its side effects of hypotension, palpitations and peripheral oedema, leading to discontinuation by most people within a relatively short time.

Post-prandial insulin secretion is stimulated by IL-1beta, which in turn is stimulated by post-prandial rises in glucose. Inhibition of IL-1beta, for example, by the IL-1 receptor antagonist anakinra, may inhibit post-prandial insulin secretion. In a double-blind, placebo controlled, randomised study, Hepprich *et al.* administered anakinra (IL-1beta receptor antagonist) or placebo to 12 people with symptomatic PBH, followed by a liquid MMT. They showed that anakinra reduced postprandial insulin secretion and subsequent hypoglycaemic events during the MMT, however there were no changes in GLP-1 or glucagon (16).

Xoma 358 (also known as RZ358) is a monoclonal antibody that is a negative allosteric modulator of the insulin receptor, it is selective to the insulin receptor and does not bind to the IGF-1 receptor. Xoma 358 was administered to 13 people with PBH in ascending dose (3 mg/kg, 6 mg/kg and 9 mg/kg). The individuals were fitted with CGM for 22 days; they underwent meal test with oral Boost (mixed meal drink) on days 3, 1, 5 and 11. The authors reported improved time to hypoglycaemia during the meal test, reduced daily periods of hypoglycaemia and correction of night-time hypoglycaemia (9 mg/kg group) on CGM (132). This medication is presently being developed for congenital hyperinsulinism and is not currently available for general use.

Treatment of PBH people in the study of Ohrstrom *et al.* with the DPP-IV inhibitor sitagliptin had an adverse effect on nadir glucose during an MMT, and the calcium channel antagonist verapamil did not appear to have any beneficial effect on hypoglycaemia (99).

In summary, there is insufficient evidence to support the treatment of PBH with IL-1beta antagonists, DPP-IV inhibitors, and calcium channel antagonists (Recommendation 3.6, Table 1).

### Call for more research into pharmacotherapeutic options

The foregoing paragraphs show that there is a dearth of rigorous clinical evidence for effective treatments in people with PBH who are refractory to dietary treatment. Such evidence is necessary not only to open treatment options but also to allow for reimbursement and funding for treatment. Hence, we call for more controlled trials of potential treatments for PBH against current standard-of-care treatment to expand the range of pharmacotherapeutic options for people with PBH (Recommendation 6.5, Table 1).

### Surgical or endoscopic options

#### Laparoscopic or endoscopic reversal or conversion surgery

Reversal or conversion surgery of various kinds has been described as solutions to obviate PBH, described mostly in retrospective patient series. In a series of five individuals (three with PBH alone, one with hypocalcaemia and one with PBH and hypocalcaemia) undergoing reversal surgery to normal anatomy or sleeve gastrectomy, three suffered post-operative complications (bleeding, gallstone pancreatitis and a trocar site infection). At a mean follow-up of 12 months, in those with PBH, there was a reduction in documented hypoglycaemic episodes and obviation of neuroglycopenic episodes. Two out of five gained weight, whereas three out of five lost further weight (133). In a series of 16 PBH individuals undergoing reversal surgery to normal anatomy ± sleeve gastrectomy after Roux-en-Y gastric bypass, gastrojejunostomy, single anastomosis duodenojejunal gastric bypass with sleeve gastrectomy and a combined vertical banded gastroplasty and distal gastric bypass, post-operative complications (colitis, leaks, intra-abdominal haemorrhage, mesenteric vein thrombosis) were described in 5 out of 16 cases. Although all individuals experienced some amelioration of PBH, 3 out of 16 continued to present with mild hypoglycaemia (2.8–3.9 mmol/L, 50–70 mg/dL). Long-term complications included gastro-oesophageal reflux in 4 out of 16, functional stenosis requiring endoscopic dilatation in 1 out of 16, anorexia in 2 out of 16 and weight regain in 7 out of 16 (134). In another small series of eight Roux-en-Y gastric bypass reversals (of which only one was described as having PBH; the others had recurrent anastomotic ulcers, recurrent nausea and vomiting and hypocalcaemia), 3 out of 8 required readmission for abdominal pain, nausea and vomiting. In the one patient with PBH, the hypoglycaemia was described as resolved after reversal. During a mean follow-up of 14 months or so, weight stability was seen in 2 out of 7 individuals, partial weight regain was seen in 4 out of 7 individuals and one regained weight to above baseline (135). Kantharia *et al.* described a case study of one patient with PBH who underwent reversal surgery leading to resolution of PBH, but accompanied by partial weight regain (136). Woods *et al.* described two cases of severe PBH who underwent surgical reversion and conversion to sleeve gastrectomy, with resolution of the PBH but no described follow-up to ascertain any tendency to weight regain (137). Davis *et al.* described a series of seven individuals who received a reversal of RYGB to sleeve gastrectomy (*n*=4) and ‘normal’ anatomy, with relief of PBH but variable weight gain after surgery (mean 16 kg, but one subject had weight gain up of 58 kg despite revision to sleeve gastrectomy) (138). Mehta *et al.* described a series of eight individuals with severe PBH after Roux-en-Y gastric bypass who underwent conversion to Roux jejuno-duodenostomy, leading to resolution of PBH in 2 out of 8, some amelioration in 4 out of 8 and minimal/no response in 2 out of 8, associated with variable post-operative weight change ranging from −29.5 to +18.2 kg over a mean follow-up of 35 months. One post-operative anastomotic bleed was managed with transfusion (139). Carter *et al.* described a series of 12 individuals undergoing laparoscopic conversion of Roux-en-Y gastric bypass to SG where 2 out of 12 had PBH and 1 had dumping (other indications included refractory marginal ulceration, stricture, dumping, gastrogastric fistula and failure to lose weight). In this series, 4 out of 12 encountered post-operative complications such as thromboembolism, haemorrhage requiring transfusion, pancreatic leak requiring re-operation, wound seroma and anastomotic leak. Six out of 12 required re-admission within 30 days of discharge, mainly for poor oral intake and nausea, with one being shown to have a stenosis at the gastro-gastric anastomosis. On average, over 15 months or so of follow-up, there was a post-operative decrease in BMI of 2.2 kg/m^2^ (−13.9 to +12.0). It was stated that the PBH and dumping syndrome resolved, with no further details (140). Another case report suggested that a laparoscopic Roux-en-Y gastric bypass reversal plus concomitant SG was effective in resolving PBH in one patient. Any post-operative weight change was not reported (141). Vilarrasa *et al.* described four individuals who underwent revisional surgery for PBH, of whom one underwent a ‘kissing operation’ with re-anastamosis alimentary limb and the antral remnant (later revised to re-anastamose the gastric pouch to the gastric remnant), two underwent conversion to normal anatomy and one resection of a ‘candy cane’ Roux limb and gastric pouch restriction. The first patient continued to have PBH which was managed with medical treatment, the others were described as having resolution of their PBH (131). Finally, in the de Heide *et al.* case series of 120 individuals with PBH, of whom five underwent Roux-en-Y gastric bypass reversal, 4 out of 5 had full resolution of their symptoms and 1 out of 5 had minimal/no resolution although weight regain between 24 and 55 kg was reported (100).

In summary, various case reports and case series utilising different techniques for the reversal/conversion of Roux-en-Y gastric bypass may offer a solution for severe PBH. However, depending on the type of procedure, these procedures may be associated with a high rate of early post-operative complications. Weight regain has been reported but appears variable in nature. We recommend that these procedures are discussed with the patient in a multidisciplinary setting involving surgeons experienced in such procedures. Full disclosure as to the likelihood of post-operative complications and weight regain is required (Recommendation 4.1, Table 1).

#### Gastric banding or silastic ring placement

In a small case series of 12 individuals, Z’Graggen and colleagues described the placement of a silastic ring or adjustable gastric band around the gastric pouch in Roux-en-Y gastric bypass and reported that this was effective in resolving PBH in 11 out of 12 participants (142). The case series by de Heide *et al.* also described 13 individuals with a ‘banded bypass’ utilising an adjustable band and gastric ring, with 9 out of 13 reported to have complete resolution (100% reduction in hypo events), 3 out of 13 experiencing a 50–100% reduction in hypos and 1 out of 13 experiencing a 0–20% reduction in hypos with minimal weight regain reported (100).

In summary, limited clinical experience suggests that restriction of the gastric pouch has some efficacy in treating PBH with relatively few complications reported. However, there is insufficient evidence to recommend this approach.

#### Endoscopic approaches

Endoscopic revision of a dilated gastro-jejunal anastomosis with transoral outlet reduction (sometimes abbreviated TORe) is an option for excessive weight regain and dumping syndrome after gastric bypass. In a retrospective review of 107 individuals, in which 26 were reported to have dumping syndrome (but no explicit PBH), this procedure was effective in resolving dumping syndrome in 90–100% (143). A case report suggested that this procedure was effective in resolving PBH in a patient after Roux-en-Y gastric bypass (144). In a case series of 11 individuals with PBH reported at a conference, this procedure was effective in resolving PBH in 10 out of 11, but 10 out of 11 experienced weight regain (on average 5.6 kg) over a relatively short follow-up period of 145 days (145). In a series of four individuals undergoing an endoscopic approach using lumen-apposing metallic stents to create a gastro-gastrostomy to reverse Roux-en-Y gastric bypass, the authors described resolution of PBH in all four but no description of any weight regain post procedure was given (146).

In summary, endoscopic approaches such as TORe show promise in resolving PBH but may be associated with weight regain, and no evidence of long-term efficacy is available. We call for research into the efficacy of these procedures in PBH (Recommendation 6.6, Table 1).

#### Pancreatectomy

Initial reports from Service *et al.* suggested that Roux-en-Y gastric bypass and PBH were associated with pancreatic nesidioblastosis (diffuse hyperplasia and hypertrophy of islet cells) in a series of six individuals, which was treated with distal pancreatectomy. They reported that in 5 out of 6 individuals, there was resolution of hypoglycaemia (147). Z’Graggen *et al.,* in addition to describing the silastic ring restriction mentioned above, also subjected three individuals to a distal pancreatectomy in addition to the gastric restriction (142). Vilarasa *et al.* described three individuals who underwent partial pancreatectomy for PBH and were reported symptom-free at follow-up 1–2 years after surgery (131). Faro *et al.* described a single case of a patient with PBH with a pancreatic tail tumour who was cured with a distal pancreatectomy together with diazoxide and hydrochlorothiazide therapy (43). However, partial pancreatectomy is associated with multiple complications, including pancreatic fistula, collection development, post-operative haemorrhage, pancreatitis, thrombosis of the portal vein and superior mesenteric vein, resection of the spleen and hyposplenism, pancreatogenic diabetes and pancreatic exocrine deficiency.

In summary, case reports show some efficacy from partial pancreatectomy in selected cases. We recommend that this is considered in cases where there is evidence of a coincident insulinoma/pancreatic mass and evidence of localised excess insulin secretion. Cases should be referred to experienced hepato-pancreato-biliary multidisciplinary teams (Recommendation 4.2, Table 1).

## Driving and PBH

PBH negatively impacts driving performance (148). Current DVLA regulations (https://www.gov.uk/guidance/diabetes-mellitus-assessing-fitness-to-drive#hypoglycaemia-due-to-other-causes, revised Jun 2022) suggest that for group 1 drivers (car and motorcycle), all of the following criteria should be met prior to licencing:

- Adequate awareness of hypoglycaemia;
- Practices appropriate glucose monitoring: glucose testing no more than 2 h before the start of the first journey and every 2 h after driving has started, with a maximum of 2 hours between the pre-driving glucose test and the first test performed after driving has started;
- Demonstrates an understanding of the risks of hypoglycaemia;
- Not regarded as a likely risk to the public when driving; and
- Remains under regular clinical review for the management of the underlying medical condition.

For group 2 drivers (bus and lorry), the following criteria needs to be met prior to licencing:

- Full awareness of hypoglycaemia;
- No episode of severe hypoglycaemia in the preceding 12 months;
- Practices appropriate glucose monitoring (see above);
- Demonstrates an understanding of the risks of hypoglycaemia;
- Not regarded as a likely risk to the public when driving; and
- Remains under regular clinical review for the management of the underlying medical condition.

With regard to the technology for glucose monitoring, it is acceptable to use intermittently scanned glucose monitoring sensors (e.g. FreeStyle Libre systems) or CGM (e.g. Medtronic or Dexcom systems) for driving group 1 vehicles, although back-up capillary blood glucose monitoring equipment should be kept in the vehicle and used to confirm sensor readings on occasions when the sensor glucose is ≤4.0 mmol/L, when there are hypoglycaemia symptoms, or if the sensor reading is not consistent with symptoms. For group 2 drivers, the sensor systems above are not allowed, and drivers must use capillary blood glucose monitoring.

If severe hypoglycaemia (defined as an episode of hypoglycaemia requiring the assistance of another person) occurs due to PBH, or if there is evidence of unawareness of hypoglycaemia, driving must stop, and the patient should notify DVLA (Recommendations 5.1–5.4 summarise this advice (Table 1)).

## Supplementary Materials

Appendix 1 Below is the search strategy for Medline conducted through the Ovid platform.

## Declaration of interest

T.M-M.T. declares that she is a shareholder in and consultant for Zihipp Ltd. B.K., C.B.L., G.A.B., R.A., S.P. and K.M. declare no conflicts of interest. S.A. declares consultancy fees for educational events from Johnson & Johnson. J.H. and K.McC. declare honoraria for speaking engagements and advisory work for Novo Nordisk. B.McG. declares that she is a shareholder in Reset Health, a research grant from Novo Nordisk, honoraria for advisory work for Novo Nordisk, Pfizer, Johnson & Johnson, Boehringer Ingleheim, and consultancy fees for educational events from Novo Nordisk, Lilly, Amgen, Astra Zeneca, Sanofi and Johnson & Johnson. S.F. collaborates with and receives funding from Novo Nordisk (Cell Therapy Programme). G.K.D. has received research grants from Novo Nordisk and DDM, as well as honoraria for lectures, presentations, speaker’s bureaus, manuscript writing and educational events from Novo Nordisk, Johnson & Johnson/Ethicon and Medtronic. R.L.B. reports honoraria from Novo Nordisk, Eli Lilly, Medscape, and ViiV Healthcare Ltd., and advisory board and consultancy work for Novo Nordisk, Eli Lilly, Pfizer, Gila Therapeutics Ltd., Epitomee Medical Ltd. and ViiV Healthcare Ltd., and from May 2023 is an employee and shareholder of Eli Lilly and Company.

## Funding

No funding was required for the development of this guidance.
