# Supplementary material for: Society for Endocrinology guidelines for the diagnosis and management of post-bariatric hypoglycaemia
Source: Endocr Connect. 2024 Apr 1;13(5):e230285. doi: 10.1530/EC-23-0285 (PMC11046333; doi:10.1530/EC-23-0285)
Supplement: Appendix 1 Below is the search strategy for Medline conducted through the Ovid platform. [file supplementary_table_1.pdf]

Appendix 1

Below is the search strategy for Medline conducted through the Ovid platform.

#	**Searches**
1	(bariatric surg$ or bariatric operation$ or bariatric procedure$ or obesity surg$).mp.
2	exp Bariatric Surgery/
3	(sleeve gastrectom$ or gastric sleev$ or laparascopic sleeve gastrectom$ or lap sleeve$ or vertical sleeve gastrectom$ or VSG).mp.
4	(Gastroplast$ or endoscopic gastroplast$ or endoscopic sleeve gastroplast$ or vertical banded gastroplast$ or vbg or banded gastroplast$).mp.
5	(LAGB or laparascopic adjust$ gastric band$ or lap band$).mp.
6	(RYGB or Roux en Y or Roux en Y gastric bypass$ or gastric bypass$ or laparascopic gastric bypass$).mp.
7	(biliopancreatic diversion$ or BPD or BPD with DS or biliopancreatic diversion with duodenal switch or biliopancreatic diversion without duodenal switch or Duodenal switch).mp.

8	(jejunoileal bypass$ or jejuno ileal bypass$ or ileojejunal bypass$ or ileo jejunal bypass$).mp.
9	(jejunoileal bypass$ or jejuno ileal bypass$ or ileojejunal bypass$ or ileo jejunal bypass$).mp.
10	(mini bypass$ or MOAGB or mono anostomo$ gastric bypass$ or OAGB or one anastomo$ gastric bypass$ or loop bypass$).mp.
11	exp Hypoglycemia/
12	hypoglyc?emia.mp.
13	(postprandial hypoglyc?emia or post prandial hypoglyc?emia or hyperinsulin?emic hypoglyc?emia or postbariatric hypoglyc?emia or post bariatric hypoglyc?emia or Whipple$ triad or dumping or late dumping).mp.
14	1 or 2 or 3 or 4 or 5 or 6 or 7 or 8 or 9 or 10
15	11 or 12 or 13
16	14 and 15
